# (*E*)-2-[4-(Dimethyl­amino)styr­yl]-1-methyl­quinolinium 4-methyl­benzene­sulfonate monohydrate^1^

**DOI:** 10.1107/S1600536808040671

**Published:** 2008-12-10

**Authors:** Thawanrat Kobkeatthawin, Thitipone Suwunwong, Suchada Chantrapromma, Hoong-Kun Fun

**Affiliations:** a Crystal Materials Research Unit, Department of Chemistry, Faculty of Science, Prince of Songkla University, Hat-Yai, Songkhla 90112, Thailand; b X-ray Crystallography Unit, School of Physics, Universiti Sains Malaysia, 11800 USM, Penang, Malaysia

## Abstract

In the title compound, C_20_H_21_N_2_^+^·C_7_H_7_O_3_S^−^·H_2_O, the cation is essentially planar, as indicated by the dihedral angle of 2.79 (13)° between the quinolinium and the dimethylaminophenyl rings, and exists in the *E* configuration. The π-conjugated planes of the cation and the anion are inclined to each other at a dihedral angle of 66.95 (12)°. The cation is linked to the anion through C—H⋯O hydrogen bonds and the anion is further linked with the water mol­ecule by O—H⋯O hydrogen bonds, forming a three-mol­ecule unit. These units are arranged in a face-to-face manner into a ribbon-like structure along the *b* axis. The ribbons are stacked along the *c* axis. The crystal structure is further stabilized by C—H⋯π inter­actions involving the dimethyl­amino­phenyl and methyl­phenyl rings. A π–π inter­action with a centroid–centroid distance of 3.6074 (19) Å is also observed.

## Related literature

For bond-length data, see: Allen *et al.* (1987 ▶). For details of hydrogen-bond motifs, see: Bernstein *et al.* (1995 ▶). For background to NLO materials research, see: Dittrich *et al.* (2003 ▶); Nogi *et al.* (2000 ▶); Ogawa *et al.* (2008 ▶); Otero *et al.* (2002 ▶); Sato *et al.* (1999 ▶); Weir *et al.* (2003 ▶); Yang *et al.* (2007 ▶). For related structures, see, for example: Adachi *et al.* (1999 ▶); Chantrapromma *et al.* (2008 ▶); Ogawa *et al.* (2008 ▶); Rahman *et al.* (2003 ▶).
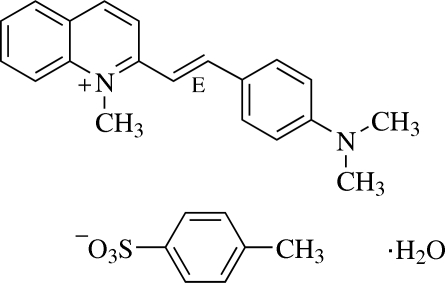


## Experimental

### 

#### Crystal data

C_20_H_21_N_2_^+^·C_7_H_7_O_3_S^−^·H_2_O
*M* *_r_* = 478.60
Triclinic, 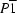

*a* = 10.9739 (5) Å
*b* = 11.1789 (5) Å
*c* = 11.1923 (9) Å
α = 97.133 (5)°
β = 100.322 (5)°
γ = 117.021 (3)°
*V* = 1169.78 (13) Å^3^
*Z* = 2
Mo *K*α radiation
μ = 0.18 mm^−1^
*T* = 100.0 (1) K
0.24 × 0.19 × 0.08 mm

#### Data collection

Bruker SMART APEX2 CCD area-detector diffractometer
Absorption correction: multi-scan (**SADABS**; Bruker, 2005 ▶) *T*_min_ = 0.958, *T*_max_ = 0.986
17557 measured reflections
5390 independent reflections
3272 reflections with *I* > 2σ(*I*)
*R*_int_ = 0.073

#### Refinement

*R*[*F* ^2^ > 2σ(*F* ^2^)] = 0.072
*wR*(*F* ^2^) = 0.203
*S* = 1.05
5390 reflections
311 parameters
H-atom parameters constrained
Δρ_max_ = 0.60 e Å^−3^
Δρ_min_ = −0.47 e Å^−3^

### 

Data collection: *APEX2* (Bruker, 2005 ▶); cell refinement: *SAINT* (Bruker, 2005 ▶); data reduction: *SAINT*; program(s) used to solve structure: *SHELXTL* (Sheldrick, 2008 ▶); program(s) used to refine structure: *SHELXTL*; molecular graphics: *SHELXTL*; software used to prepare material for publication: *SHELXTL* and *PLATON* (Spek, 2003 ▶).

## Supplementary Material

Crystal structure: contains datablocks global, I. DOI: 10.1107/S1600536808040671/is2369sup1.cif

Structure factors: contains datablocks I. DOI: 10.1107/S1600536808040671/is2369Isup2.hkl

Additional supplementary materials:  crystallographic information; 3D view; checkCIF report

## Figures and Tables

**Table 1:** Hydrogen-bond geometry (Å, °)

*D*—H⋯*A*	*D*—H	H⋯*A*	*D*⋯*A*	*D*—H⋯*A*
O1*W*—H1*W*⋯O2	0.90	1.96	2.839 (4)	164
O1*W*—H2*W*⋯O1^i^	0.88	2.01	2.893 (3)	179
C10—H10*A*⋯O3^ii^	0.93	2.57	3.460 (4)	162
C17—H17*A*⋯O3^ii^	0.93	2.45	3.344 (5)	163
C20—H20*A*⋯O3^ii^	0.96	2.33	3.204 (6)	151
C20—H20*B*⋯O1^iii^	0.96	2.49	3.388 (4)	156
C26—H26*A*⋯O2	0.93	2.51	2.884 (5)	104
C7—H7*A*⋯*Cg*4^iv^	0.93	2.97	3.615 (4)	128
C23—H23*A*⋯*Cg*3^v^	0.93	2.82	3.594 (4)	141

Symmetry codes: (i) 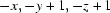; (ii) 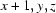; (iii) 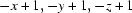; (iv) 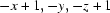; (v) 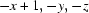. *Cg*3 and *Cg*4 are the centroids of the C12–C17 and C21–C26 rings, respectively.

**Fig. 1.**
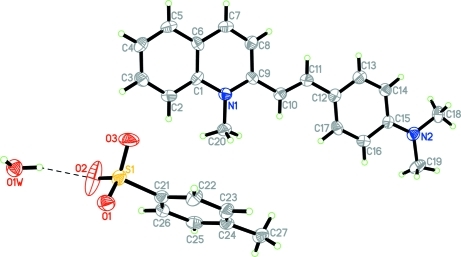
The asymmetric unit of (I), showing 50% probability displacement ellipsoids and the atom-numbering scheme.

**Fig. 2.**
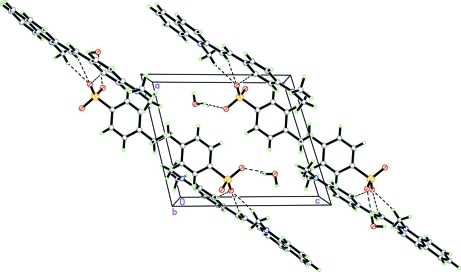
The crystal packing of (I) viewed along the b axis. The O—H···O and weak C—H···O interactions are drawn as dashed lines.

## supplementary crystallographic information

### Comment

There has been considerable interest in organic nonlinear optical materials that could be used for applications including telecommunications, optical computing and optical data storage. Organic crystals with extensive conjugated π systems are attractive candidates for nonlinear optic (NLO) studies because of their large hyperpolariability (β) and ease of preparation (Dittrich *et al.*, 2003; Nogi *et al.*, 2000; Ogawa *et al.*, 2008; Otero *et al.*, 2002; Sato *et al.*, 1999; Weir *et al.*, 2003; Yang *et al.*, 2007). 4-*N*,*N*-dimethylamino-4'-*N*'-methyl-stilbazolium tosylate (DAST) is one of the promising NLO material (Adachi *et al.*, 1999). Previous studies (Dittrich *et al.*, 2003; Nogi *et al.*, 2000; Sato *et al.*, 1999) have shown that the DAST and its analogues exhibit second-order non-linear optical properties. One strategy to enhance the hyperpolariability of the cations is by elongation of its π-conjugation system. Based on these previous studies, we have synthesized the title compound which was designed by increasing the π-conjugate system with the replacement of the cationic pyridinium ring that is present in DAST by the quinolinium ring. The crystal structure of the title compound is reported in this study.

Figure 1 shows the asymmetric unit of the title compound (I) which consists of a C_20_H_21_N_2_^+^ cation, a C_7_H_7_O_3_S^-^ anion and one H_2_O molecule. The cation exists in the *E* configuration with respect to the C10═C11 double bond [1.328 (4) Å, the corresponding value is 1.357 (2) Å in Chantrapromma *et al.*, 2008]. The cation molecule is essentially planar as indicated by the dihedral angle between the quinolinium and the dimethylaminophenyl rings being 2.79 (13)° [9.26 (6) ° in Chantrapromma *et al.*, 2008] with the torsion angles C8–C9–C10–C11 = -0.1 (5)° and C10–C11–C12–C17 = 2.4 (5)°. Both methyl groups of dimethylamino moiety are slightly twisted from the mean plane of the attached C12–C17 ring as indicated by the torsion angle C18—N2–C15–C14 = 3.6 (4)° and C19–N2–C15–C16 = -6.3 (4)°. The relative arrangement of cation and anion is shown by the interplanar angles between the mean plane of the 4-methylphenyl ring and those of the quinolinium and dimethylaminophenyl system which are 67.80 (13) and 67.19 (16)°, respectively. Besides the O—H···O hydrogen bonded to water molecule, the atom O2 of the sulfonate also contributed to a weak intramolecular C26—H26A···O2 interaction (Table 1) forming an S(5) ring motif (Bernstein *et al.*, 1995). The bond lengths (Allen *et al.*, 1987) and angles in (I) are in normal ranges and comparable with a related structure (Chantrapromma *et al.*, 2008).

In the crystal packing, all O atoms of the sulfonate group are involved in weak C—H···O interactions (Table 1). The cation is linked to the anion by weak C—H···O interactions and the anion is further linked to the water molecule by O—H···O hydrogen bonds, forming a three-molecule unit (Table 1 and Fig. 2). These three-molecule units are arranged in a face-to-face manner into a ribbon-like structure along the *b* axis and these ribbons are stacked along the *c* axis (Fig. 2). The crystal structure is further stabilized by C—H···π interactions (Table 1). A π–π interaction with the distance *Cg*_1_···*Cg*_2_^iv^ = 3.6074 (19) Å [symmetry code: (iv) 1 - *x*, -*y*, 1 - *z*] is observed; *Cg*_1_, *Cg*_2_, *Cg*_3_ and *Cg*_4_ are the centroids of the N1/C1/C6–C9, C1–C6, C12–C17 and C21–C26 rings, respectively.

### Experimental

(*E*)-2-[4-(Dimethylamino)styryl]-1-methylpyridinium iodide (compound A) was synthesized according to our previously reported procedure (Chantrapromma *et al.*, 2008). Silver(I) *p*-toluensulfonate (compound B) was synthesized according to a previous method (Rahman *et al.*, 2003). The title compound was then prepared by mixing compound A (0.20 g, 0.5 mmol) in hot methanol (50 ml) and compound B (0.12 g, 0.5 mmol) in hot methanol (30 ml). The mixture immediately yielded a grey precipitate of silver iodide. After stirring the mixture for 30 min, the precipitate of silver iodide was removed and the resulting solution was evaporated yielding a green solid. Green block-shaped single crystals of the title compound suitable for X-ray structure determination were recrystalized from methanol by slow evaporation of the solvent at room temperature a few weeks (m.p. 557–558 K).

### Refinement

All H atoms were placed in calculated positions, with d(O—H) = 0.88–0.90 Å, *U*_iso_(H) = 1.5*U*_eq_(O), d(C—H) = 0.93 Å, *U*_iso_(H) = 1.2*U*_eq_(C) for aromatic and CH, and 0.96 Å, *U*_iso_(H) = 1.5*U*_eq_(C) for CH_3_ atoms. A rotating group model was used for the methyl groups. The highest residual electron density peak is located at 0.98 Å from C8 and the deepest hole is located at 0.96 Å from S1.

### Crystal data

**Table:**

C_20_H_21_N_2_^+^·C_7_H_7_O_3_S^−^·H_2_O	*Z* = 2
*M**_r_* = 478.60	*F*(000) = 508
Triclinic, *P*1	*D*_x_ = 1.359 Mg m^−^^3^
Hall symbol: -P 1	Melting point = 557–558 K
*a* = 10.9739 (5) Å	Mo *K*α radiation, λ = 0.71073 Å
*b* = 11.1789 (5) Å	Cell parameters from 5390 reflections
*c* = 11.1923 (9) Å	θ = 1.2–27.5°
α = 97.133 (5)°	µ = 0.18 mm^−^^1^
β = 100.322 (5)°	*T* = 100 K
γ = 117.021 (3)°	Block, green
*V* = 1169.78 (13) Å^3^	0.24 × 0.19 × 0.08 mm

### Data collection

**Table:**

Bruker SMART APEX2 CCD area-detector diffractometer	5390 independent reflections
Radiation source: fine-focus sealed tube	3272 reflections with *I* > 2σ(*I*)
graphite	*R*_int_ = 0.073
Detector resolution: 8.33 pixels mm^-1^	θ_max_ = 27.5°, θ_min_ = 1.9°
ω scans	*h* = −14→14
Absorption correction: multi-scan (*SADABS*; Bruker, 2005)	*k* = −14→14
*T*_min_ = 0.958, *T*_max_ = 0.986	*l* = −14→11
17557 measured reflections	

### Refinement

**Table:**

Refinement on *F*^2^	Primary atom site location: structure-invariant direct methods
Least-squares matrix: full	Secondary atom site location: difference Fourier map
*R*[*F*^2^ > 2σ(*F*^2^)] = 0.072	Hydrogen site location: inferred from neighbouring sites
*wR*(*F*^2^) = 0.203	H-atom parameters constrained
*S* = 1.05	*w* = 1/[σ^2^(*F*_o_^2^) + (0.0951*P*)^2^ + 0.1631*P*] where *P* = (*F*_o_^2^ + 2*F*_c_^2^)/3
5390 reflections	(Δ/σ)_max_ < 0.001
311 parameters	Δρ_max_ = 0.60 e Å^−^^3^
0 restraints	Δρ_min_ = −0.47 e Å^−^^3^

### Special details

**Table:**

Experimental. The low-temparture data was collected with the Oxford Cryosystem Cobra low-temperature attachment.
Geometry. All e.s.d.'s (except the e.s.d. in the dihedral angle between two l.s. planes) are estimated using the full covariance matrix. The cell e.s.d.'s are taken into account individually in the estimation of e.s.d.'s in distances, angles and torsion angles; correlations between e.s.d.'s in cell parameters are only used when they are defined by crystal symmetry. An approximate (isotropic) treatment of cell e.s.d.'s is used for estimating e.s.d.'s involving l.s. planes.
Refinement. Refinement of *F*^2^ against ALL reflections. The weighted *R*-factor *wR* and goodness of fit *S* are based on *F*^2^, conventional *R*-factors *R* are based on *F*, with *F* set to zero for negative *F*^2^. The threshold expression of *F*^2^ > σ(*F*^2^) is used only for calculating *R*-factors(gt) *etc*. and is not relevant to the choice of reflections for refinement. *R*-factors based on *F*^2^ are statistically about twice as large as those based on *F*, and *R*- factors based on ALL data will be even larger.

### Fractional atomic coordinates and isotropic or equivalent isotropic displacement parameters (Å^2^)

**Table:**

	*x*	*y*	*z*	*U*_iso_*/*U*_eq_	
S1	0.16105 (8)	0.41169 (9)	0.37784 (7)	0.0306 (2)	
O1	0.0863 (2)	0.4772 (2)	0.3214 (2)	0.0361 (6)	
O2	0.2576 (3)	0.4944 (4)	0.4973 (2)	0.0784 (11)	
O3	0.0690 (3)	0.2712 (3)	0.3786 (3)	0.0708 (10)	
O1W	0.2135 (2)	0.6205 (2)	0.7095 (2)	0.0371 (6)	
H1W	0.2122	0.5660	0.6424	0.056*	
H2W	0.1227	0.5918	0.7009	0.056*	
N1	0.7361 (3)	0.0767 (3)	0.5500 (2)	0.0286 (6)	
N2	1.1460 (3)	−0.1954 (3)	0.0336 (2)	0.0342 (6)	
C1	0.6510 (3)	0.0789 (3)	0.6300 (3)	0.0270 (7)	
C2	0.6415 (3)	0.1968 (3)	0.6702 (3)	0.0331 (7)	
H2A	0.6928	0.2775	0.6453	0.040*	
C3	0.5566 (3)	0.1928 (3)	0.7459 (3)	0.0343 (8)	
H3A	0.5517	0.2722	0.7728	0.041*	
C4	0.4776 (3)	0.0760 (4)	0.7845 (3)	0.0335 (8)	
H4A	0.4200	0.0770	0.8359	0.040*	
C5	0.4840 (3)	−0.0424 (3)	0.7467 (3)	0.0332 (8)	
H5A	0.4311	−0.1218	0.7726	0.040*	
C6	0.5721 (3)	−0.0429 (3)	0.6678 (3)	0.0283 (7)	
C7	0.5835 (3)	−0.1616 (3)	0.6271 (3)	0.0333 (7)	
H7A	0.5333	−0.2419	0.6529	0.040*	
C8	0.6659 (3)	−0.1590 (3)	0.5517 (3)	0.0321 (7)	
H8A	0.6733	−0.2375	0.5274	0.039*	
C9	0.7441 (3)	−0.0373 (3)	0.5067 (3)	0.0252 (6)	
C10	0.8232 (3)	−0.0412 (3)	0.4199 (3)	0.0281 (7)	
H10A	0.8713	0.0395	0.3939	0.034*	
C11	0.8347 (3)	−0.1494 (3)	0.3726 (3)	0.0310 (7)	
H11A	0.7869	−0.2295	0.3996	0.037*	
C12	0.9139 (3)	−0.1564 (3)	0.2832 (3)	0.0297 (7)	
C13	0.9132 (3)	−0.2798 (3)	0.2436 (3)	0.0335 (7)	
H13A	0.8599	−0.3560	0.2733	0.040*	
C14	0.9879 (3)	−0.2927 (3)	0.1630 (3)	0.0304 (7)	
H14A	0.9845	−0.3772	0.1394	0.036*	
C15	1.0705 (3)	−0.1817 (3)	0.1142 (3)	0.0265 (7)	
C16	1.0722 (3)	−0.0549 (3)	0.1534 (3)	0.0296 (7)	
H16A	1.1246	0.0212	0.1232	0.035*	
C17	0.9956 (3)	−0.0444 (3)	0.2368 (3)	0.0303 (7)	
H17A	0.9988	0.0396	0.2624	0.036*	
C18	1.1368 (4)	−0.3274 (4)	−0.0096 (3)	0.0400 (8)	
H18A	1.1633	−0.3597	0.0609	0.060*	
H18B	1.1998	−0.3172	−0.0617	0.060*	
H18C	1.0412	−0.3928	−0.0566	0.060*	
C19	1.2199 (4)	−0.0842 (4)	−0.0251 (3)	0.0456 (9)	
H19A	1.2955	−0.0062	0.0374	0.068*	
H19B	1.1546	−0.0580	−0.0667	0.068*	
H19C	1.2584	−0.1150	−0.0849	0.068*	
C20	0.8178 (4)	0.2048 (3)	0.5119 (3)	0.0373 (8)	
H20A	0.8989	0.2051	0.4907	0.056*	
H20B	0.8488	0.2828	0.5794	0.056*	
H20C	0.7590	0.2104	0.4405	0.056*	
C21	0.2694 (3)	0.4028 (3)	0.2798 (3)	0.0255 (6)	
C22	0.2061 (3)	0.3196 (3)	0.1602 (3)	0.0333 (7)	
H22A	0.1077	0.2714	0.1305	0.040*	
C23	0.2884 (3)	0.3080 (3)	0.0850 (3)	0.0313 (7)	
H23A	0.2447	0.2519	0.0046	0.038*	
C24	0.4360 (3)	0.3785 (3)	0.1271 (3)	0.0291 (7)	
C25	0.4974 (3)	0.4628 (3)	0.2463 (3)	0.0300 (7)	
H25A	0.5958	0.5115	0.2759	0.036*	
C26	0.4160 (3)	0.4765 (3)	0.3227 (3)	0.0286 (7)	
H26A	0.4595	0.5349	0.4021	0.034*	
C27	0.5253 (4)	0.3608 (4)	0.0459 (3)	0.0370 (8)	
H27A	0.4763	0.3403	−0.0404	0.055*	
H27B	0.5420	0.2863	0.0616	0.055*	
H27C	0.6143	0.4446	0.0649	0.055*	

### Atomic displacement parameters (Å^2^)

**Table:**

	*U*^11^	*U*^22^	*U*^33^	*U*^12^	*U*^13^	*U*^23^
S1	0.0328 (4)	0.0371 (5)	0.0331 (4)	0.0226 (4)	0.0159 (3)	0.0132 (4)
O1	0.0380 (13)	0.0384 (13)	0.0485 (14)	0.0270 (12)	0.0196 (11)	0.0202 (11)
O2	0.0542 (18)	0.157 (3)	0.0318 (15)	0.068 (2)	0.0054 (13)	−0.0097 (17)
O3	0.095 (2)	0.0431 (16)	0.127 (3)	0.0462 (17)	0.094 (2)	0.0523 (17)
O1W	0.0345 (12)	0.0385 (14)	0.0405 (13)	0.0194 (11)	0.0115 (11)	0.0084 (11)
N1	0.0282 (14)	0.0278 (14)	0.0312 (14)	0.0141 (12)	0.0091 (11)	0.0086 (11)
N2	0.0324 (15)	0.0387 (16)	0.0352 (15)	0.0182 (14)	0.0150 (12)	0.0091 (13)
C1	0.0221 (15)	0.0381 (18)	0.0213 (15)	0.0142 (14)	0.0073 (12)	0.0086 (13)
C2	0.0332 (18)	0.0310 (18)	0.0383 (18)	0.0153 (15)	0.0120 (15)	0.0163 (15)
C3	0.0381 (18)	0.0316 (18)	0.0392 (19)	0.0222 (16)	0.0089 (15)	0.0096 (15)
C4	0.0285 (17)	0.044 (2)	0.0311 (17)	0.0186 (16)	0.0121 (14)	0.0109 (15)
C5	0.0296 (17)	0.0341 (18)	0.0315 (17)	0.0096 (15)	0.0088 (14)	0.0161 (14)
C6	0.0322 (17)	0.0255 (16)	0.0257 (16)	0.0167 (14)	−0.0004 (13)	0.0022 (12)
C7	0.0331 (17)	0.0292 (18)	0.0358 (18)	0.0116 (15)	0.0110 (15)	0.0145 (14)
C8	0.0316 (17)	0.0367 (19)	0.0280 (16)	0.0170 (16)	0.0075 (14)	0.0070 (14)
C9	0.0240 (15)	0.0229 (16)	0.0263 (16)	0.0122 (13)	0.0002 (12)	0.0035 (12)
C10	0.0259 (16)	0.0324 (18)	0.0286 (16)	0.0153 (15)	0.0085 (13)	0.0093 (13)
C11	0.0311 (17)	0.0281 (17)	0.0328 (17)	0.0130 (15)	0.0092 (14)	0.0084 (14)
C12	0.0270 (16)	0.0349 (18)	0.0266 (16)	0.0157 (15)	0.0045 (13)	0.0062 (14)
C13	0.0283 (17)	0.0358 (19)	0.0319 (17)	0.0125 (15)	0.0066 (14)	0.0080 (14)
C14	0.0284 (16)	0.0308 (18)	0.0344 (17)	0.0161 (15)	0.0106 (14)	0.0065 (14)
C15	0.0231 (15)	0.0303 (17)	0.0247 (15)	0.0135 (14)	0.0040 (12)	0.0038 (13)
C16	0.0258 (16)	0.0288 (17)	0.0328 (17)	0.0123 (14)	0.0058 (13)	0.0098 (14)
C17	0.0325 (17)	0.0285 (17)	0.0297 (17)	0.0188 (15)	0.0007 (13)	0.0008 (13)
C18	0.040 (2)	0.049 (2)	0.039 (2)	0.0293 (19)	0.0136 (16)	0.0030 (16)
C19	0.041 (2)	0.060 (3)	0.045 (2)	0.024 (2)	0.0236 (17)	0.0249 (19)
C20	0.0404 (19)	0.0348 (19)	0.044 (2)	0.0187 (17)	0.0220 (16)	0.0160 (16)
C21	0.0289 (16)	0.0276 (17)	0.0267 (15)	0.0172 (14)	0.0103 (13)	0.0113 (13)
C22	0.0266 (16)	0.043 (2)	0.0309 (17)	0.0165 (16)	0.0082 (14)	0.0102 (15)
C23	0.0352 (18)	0.0352 (18)	0.0227 (16)	0.0169 (16)	0.0069 (14)	0.0065 (13)
C24	0.0361 (17)	0.0287 (17)	0.0334 (17)	0.0207 (15)	0.0148 (14)	0.0157 (14)
C25	0.0222 (15)	0.0281 (17)	0.0388 (18)	0.0102 (14)	0.0078 (13)	0.0132 (14)
C26	0.0297 (16)	0.0240 (16)	0.0303 (16)	0.0115 (14)	0.0086 (13)	0.0063 (13)
C27	0.0410 (19)	0.045 (2)	0.042 (2)	0.0286 (18)	0.0240 (16)	0.0208 (16)

### Geometric parameters (Å, °)

**Table:**

S1—O3	1.433 (3)	C12—C17	1.396 (4)
S1—O2	1.435 (3)	C13—C14	1.359 (4)
S1—O1	1.443 (2)	C13—H13A	0.9300
S1—C21	1.782 (3)	C14—C15	1.408 (4)
O1W—H1W	0.8997	C14—H14A	0.9300
O1W—H2W	0.8784	C15—C16	1.421 (4)
N1—C9	1.353 (4)	C16—C17	1.392 (4)
N1—C1	1.411 (4)	C16—H16A	0.9300
N1—C20	1.470 (4)	C17—H17A	0.9300
N2—C15	1.368 (4)	C18—H18A	0.9600
N2—C18	1.446 (4)	C18—H18B	0.9600
N2—C19	1.453 (4)	C18—H18C	0.9600
C1—C2	1.395 (4)	C19—H19A	0.9600
C1—C6	1.409 (4)	C19—H19B	0.9600
C2—C3	1.358 (4)	C19—H19C	0.9600
C2—H2A	0.9300	C20—H20A	0.9600
C3—C4	1.374 (4)	C20—H20B	0.9600
C3—H3A	0.9300	C20—H20C	0.9600
C4—C5	1.375 (5)	C21—C22	1.382 (4)
C4—H4A	0.9300	C21—C26	1.385 (4)
C5—C6	1.423 (4)	C22—C23	1.376 (4)
C5—H5A	0.9300	C22—H22A	0.9300
C6—C7	1.416 (4)	C23—C24	1.393 (4)
C7—C8	1.336 (4)	C23—H23A	0.9300
C7—H7A	0.9300	C24—C25	1.382 (4)
C8—C9	1.451 (4)	C24—C27	1.509 (4)
C8—H8A	0.9300	C25—C26	1.385 (4)
C9—C10	1.423 (4)	C25—H25A	0.9300
C10—C11	1.328 (4)	C26—H26A	0.9300
C10—H10A	0.9300	C27—H27A	0.9600
C11—C12	1.455 (4)	C27—H27B	0.9600
C11—H11A	0.9300	C27—H27C	0.9600
C12—C13	1.392 (4)		
			
O3—S1—O2	113.8 (2)	C13—C14—H14A	119.1
O3—S1—O1	113.16 (16)	C15—C14—H14A	119.1
O2—S1—O1	112.01 (18)	N2—C15—C14	121.4 (3)
O3—S1—C21	105.11 (14)	N2—C15—C16	121.8 (3)
O2—S1—C21	105.52 (15)	C14—C15—C16	116.8 (3)
O1—S1—C21	106.33 (13)	C17—C16—C15	120.1 (3)
H1W—O1W—H2W	102.3	C17—C16—H16A	119.9
C9—N1—C1	123.0 (3)	C15—C16—H16A	119.9
C9—N1—C20	119.4 (3)	C16—C17—C12	121.9 (3)
C1—N1—C20	117.6 (2)	C16—C17—H17A	119.0
C15—N2—C18	120.6 (3)	C12—C17—H17A	119.0
C15—N2—C19	120.8 (3)	N2—C18—H18A	109.5
C18—N2—C19	117.9 (3)	N2—C18—H18B	109.5
C2—C1—C6	119.8 (3)	H18A—C18—H18B	109.5
C2—C1—N1	121.8 (3)	N2—C18—H18C	109.5
C6—C1—N1	118.4 (3)	H18A—C18—H18C	109.5
C3—C2—C1	119.4 (3)	H18B—C18—H18C	109.5
C3—C2—H2A	120.3	N2—C19—H19A	109.5
C1—C2—H2A	120.3	N2—C19—H19B	109.5
C2—C3—C4	122.7 (3)	H19A—C19—H19B	109.5
C2—C3—H3A	118.7	N2—C19—H19C	109.5
C4—C3—H3A	118.7	H19A—C19—H19C	109.5
C3—C4—C5	119.6 (3)	H19B—C19—H19C	109.5
C3—C4—H4A	120.2	N1—C20—H20A	109.5
C5—C4—H4A	120.2	N1—C20—H20B	109.5
C4—C5—C6	119.7 (3)	H20A—C20—H20B	109.5
C4—C5—H5A	120.1	N1—C20—H20C	109.5
C6—C5—H5A	120.1	H20A—C20—H20C	109.5
C1—C6—C7	119.2 (3)	H20B—C20—H20C	109.5
C1—C6—C5	118.8 (3)	C22—C21—C26	119.7 (3)
C7—C6—C5	122.0 (3)	C22—C21—S1	119.5 (2)
C8—C7—C6	120.4 (3)	C26—C21—S1	120.8 (2)
C8—C7—H7A	119.8	C23—C22—C21	120.1 (3)
C6—C7—H7A	119.8	C23—C22—H22A	120.0
C7—C8—C9	121.9 (3)	C21—C22—H22A	120.0
C7—C8—H8A	119.0	C22—C23—C24	121.2 (3)
C9—C8—H8A	119.0	C22—C23—H23A	119.4
N1—C9—C10	122.7 (3)	C24—C23—H23A	119.4
N1—C9—C8	116.9 (3)	C25—C24—C23	117.8 (3)
C10—C9—C8	120.4 (3)	C25—C24—C27	121.3 (3)
C11—C10—C9	126.0 (3)	C23—C24—C27	120.9 (3)
C11—C10—H10A	117.0	C24—C25—C26	121.6 (3)
C9—C10—H10A	117.0	C24—C25—H25A	119.2
C10—C11—C12	127.1 (3)	C26—C25—H25A	119.2
C10—C11—H11A	116.4	C21—C26—C25	119.5 (3)
C12—C11—H11A	116.4	C21—C26—H26A	120.3
C13—C12—C17	117.2 (3)	C25—C26—H26A	120.3
C13—C12—C11	119.0 (3)	C24—C27—H27A	109.5
C17—C12—C11	123.8 (3)	C24—C27—H27B	109.5
C14—C13—C12	122.2 (3)	H27A—C27—H27B	109.5
C14—C13—H13A	118.9	C24—C27—H27C	109.5
C12—C13—H13A	118.9	H27A—C27—H27C	109.5
C13—C14—C15	121.9 (3)	H27B—C27—H27C	109.5
			
C9—N1—C1—C2	−178.0 (3)	C11—C12—C13—C14	−178.1 (3)
C20—N1—C1—C2	1.2 (4)	C12—C13—C14—C15	−0.2 (5)
C9—N1—C1—C6	0.9 (4)	C18—N2—C15—C14	3.6 (4)
C20—N1—C1—C6	−179.9 (3)	C19—N2—C15—C14	174.2 (3)
C6—C1—C2—C3	0.3 (4)	C18—N2—C15—C16	−176.9 (3)
N1—C1—C2—C3	179.2 (3)	C19—N2—C15—C16	−6.3 (4)
C1—C2—C3—C4	−0.6 (5)	C13—C14—C15—N2	179.6 (3)
C2—C3—C4—C5	0.5 (5)	C13—C14—C15—C16	0.1 (4)
C3—C4—C5—C6	−0.2 (5)	N2—C15—C16—C17	−179.1 (3)
C2—C1—C6—C7	−179.6 (3)	C14—C15—C16—C17	0.4 (4)
N1—C1—C6—C7	1.5 (4)	C15—C16—C17—C12	−0.9 (4)
C2—C1—C6—C5	0.0 (4)	C13—C12—C17—C16	0.7 (4)
N1—C1—C6—C5	−178.9 (3)	C11—C12—C17—C16	178.6 (3)
C4—C5—C6—C1	−0.1 (4)	O3—S1—C21—C22	53.9 (3)
C4—C5—C6—C7	179.5 (3)	O2—S1—C21—C22	174.5 (3)
C1—C6—C7—C8	−1.3 (4)	O1—S1—C21—C22	−66.3 (3)
C5—C6—C7—C8	179.2 (3)	O3—S1—C21—C26	−125.2 (3)
C6—C7—C8—C9	−1.3 (5)	O2—S1—C21—C26	−4.6 (3)
C1—N1—C9—C10	176.0 (3)	O1—S1—C21—C26	114.6 (2)
C20—N1—C9—C10	−3.3 (4)	C26—C21—C22—C23	1.4 (5)
C1—N1—C9—C8	−3.3 (4)	S1—C21—C22—C23	−177.7 (2)
C20—N1—C9—C8	177.5 (3)	C21—C22—C23—C24	0.2 (5)
C7—C8—C9—N1	3.5 (4)	C22—C23—C24—C25	−1.1 (5)
C7—C8—C9—C10	−175.8 (3)	C22—C23—C24—C27	177.6 (3)
N1—C9—C10—C11	−179.3 (3)	C23—C24—C25—C26	0.6 (4)
C8—C9—C10—C11	−0.1 (5)	C27—C24—C25—C26	−178.1 (3)
C9—C10—C11—C12	179.5 (3)	C22—C21—C26—C25	−1.9 (4)
C10—C11—C12—C13	−179.7 (3)	S1—C21—C26—C25	177.2 (2)
C10—C11—C12—C17	2.4 (5)	C24—C25—C26—C21	0.9 (4)
C17—C12—C13—C14	−0.2 (5)		

### Hydrogen-bond geometry (Å, °)

**Table:**

*D*—H···*A*	*D*—H	H···*A*	*D*···*A*	*D*—H···*A*
O1W—H1W···O2	0.90	1.96	2.839 (4)	164
O1W—H2W···O1^i^	0.88	2.01	2.893 (3)	179
C10—H10A···O3^ii^	0.93	2.57	3.460 (4)	162
C17—H17A···O3^ii^	0.93	2.45	3.344 (5)	163
C20—H20A···O3^ii^	0.96	2.33	3.204 (6)	151
C20—H20B···O1^iii^	0.96	2.49	3.388 (4)	156
C26—H26A···O2	0.93	2.51	2.884 (5)	104
C7—H7A···Cg4^iv^	0.93	2.97	3.615 (4)	128
C23—H23A···Cg3^v^	0.93	2.82	3.594 (4)	141

Symmetry codes:  (i) −*x*, −*y*+1, −*z*+1; (ii) *x*+1, *y*, *z*; (iii) −*x*+1, −*y*+1, −*z*+1; (iv) −*x*+1, −*y*, −*z*+1; (v) −*x*+1, −*y*, −*z*.
